# Variable selection methods for identifying predictor interactions in data with repeatedly measured binary outcomes

**DOI:** 10.1017/cts.2020.556

**Published:** 2020-11-16

**Authors:** Bethany J. Wolf, Yunyun Jiang, Sylvia H. Wilson, Jim C. Oates

**Affiliations:** 1 Department of Public Health Sciences, Medical University of South Carolina, Charleston, SC, USA; 2 Milken Institute School of Public Health, Biostatistics Center, George Washington University, Rockville, MD, USA; 3 Department of Anesthesia and Perioperative Medicine, Medical University of South Carolina, Charleston, SC, USA; 4 Department of Medicine, Division of Rheumatology and Immunology, Medical University of South Carolina, Charleston, SC, USA

**Keywords:** Variable selection, interactions, penalized regression, boosting, two-stage algorithm

## Abstract

**Introduction:**

Identifying predictors of patient outcomes evaluated over time may require modeling interactions among variables while addressing within-subject correlation. Generalized linear mixed models (GLMMs) and generalized estimating equations (GEEs) address within-subject correlation, but identifying interactions can be difficult if not hypothesized *a priori*. We evaluate the performance of several variable selection approaches for clustered binary outcomes to provide guidance for choosing between the methods.

**Methods:**

We conducted simulations comparing stepwise selection, penalized GLMM, boosted GLMM, and boosted GEE for variable selection considering main effects and two-way interactions in data with repeatedly measured binary outcomes and evaluate a two-stage approach to reduce bias and error in parameter estimates. We compared these approaches in real data applications: hypothermia during surgery and treatment response in lupus nephritis.

**Results:**

Penalized and boosted approaches recovered correct predictors and interactions more frequently than stepwise selection. Penalized GLMM recovered correct predictors more often than boosting, but included many spurious predictors. Boosted GLMM yielded parsimonious models and identified correct predictors well at large sample and effect sizes, but required excessive computation time. Boosted GEE was computationally efficient and selected relatively parsimonious models, offering a compromise between computation and parsimony. The two-stage approach reduced the bias and error in regression parameters in all approaches.

**Conclusion:**

Penalized and boosted approaches are effective for variable selection in data with clustered binary outcomes. The two-stage approach reduces bias and error and should be applied regardless of method. We provide guidance for choosing the most appropriate method in real applications.

## Introduction

It has been hypothesized that many common diseases result from interactions among genetic, clinical, and environmental factors [1–4]. Identifying predictors of patients’ disease status may necessitate modeling interactions among predictors [5, 6]. Clinical studies also often evaluate patient outcomes over time requiring methods that can account for within-subject correlation. Generalized linear mixed model (GLMM) and generalized estimating equations (GEEs) address the correlation between repeated measures collected on a patient within a linear model framework [7]. GLMMs incorporate a subject-specific random effect to account for correlation between outcome measures on the same patients, while GEEs account for this correlation in the residual variance matrix. The random subject effect in the GLMM allows for subject-specific inference, and fixed effects are interpreted conditional on the subject-specific effect, while GEEs yield a marginal model focused on the average population response given the covariates. Although both GLMMs and GEEs can model interactions, interactions should be specified *a priori* and sufficient sample size must be available to include interactions and main effects [8]. In exploratory studies, interactions are not generally hypothesized *a priori*, and failure to examine interactions may result in failure to identify predictors associated with the outcome. In such cases, application of variable selection techniques can be employed to identify main effects and interactions most strongly associated with the outcome.

Methods for selecting variables in regression models fall into two categories: traditional stepwise variable selection methods and sparse model solutions. Traditional variable selection approaches include forward, backward, and stepwise algorithms that select variables for inclusion in a model based on statistical significance or measures of model fit. However, such approaches yield unstable parameter estimates, particularly in data with correlated predictors or outcomes [9, 10].

Sparse model solutions simultaneously evaluate all potential covariates and shrink regression coefficient estimates toward zero. Penalized regression techniques penalize coefficients the further their estimated value is from zero by including a penalty term in the likelihood. Lasso regression, a popular penalized regression approach, includes the sum of the absolute values of the regression coefficients in the likelihood (referred to as the *L*_1_ penalty) which shrinks many coefficients to zero providing a means of variable selection [11]. Schelldorfer et al. [12] extended lasso to the GLMM setting, a method referred to as glmmLasso. Boosting, originally proposed to improve classification procedures by combining estimates based on iteratively reweighted residuals [13, 14], can also be used for variable selection by applying a component-wise approach [15]. Tutz and Groll [16] extend this component-wise boosting approach to the GLMM setting, a method referred to as GMMBoost. The GEE approach offers an alternative for modeling correlated binary outcomes without focusing on the within-subject covariance structure by estimating the population-average responses using a marginal approach. Similar to GMMBoost, Wolfson [17] developed a boosted estimating equation approach, EEBoost, for variable selection based on minimizing the *L*_1_-constrained projected likelihood ratio. Brown et al. [18] further extend EEBoost to the GEE setting. The glmmLasso, GMMBoost, and GEEBoost methods provide a means of variable selection in data with large number of predictors and repeated measures in order to achieve a sparse model. Of note, all three methods yield biased estimates of the regression parameters [15, 19, 20]. In order to address bias in parameter estimates, one could use a two-stage approach in which predictors are selected in the first stage using a sparse modeling approach followed by refitting a GLMM (or GEE) using the predictors selected in the first stage. However, such an approach has not been evaluated using sparse model solutions in data with repeatedly measured binary outcomes.

The goals of this paper are to provide an overview of variable selection methods for data with repeatedly measured binary outcomes and to evaluate the performance of each approach for recovery of interactions and main effects associated an outcomes to provide guidance for choosing from among these methods in application. We present a simulation evaluating the relative performance of stepwise selection, glmmLasso, GMMBoost, and GEEBoost for correct identification of nonzero regression parameters, model parsimony, and bias in the regression parameters. Additionally, we examine the ability of a two-stage approach to reduce bias in regression estimates from the three penalized and boosting methods. We apply each method to two clinical data sets and explore differences in the models returned by each method. The first data set examines a set of protein biomarkers for predicting treatment response in lupus nephritis. The second explores clinical and patient characteristics associated with risk of hypothermia over time in hip or knee arthroplasty patients. We conclude by summarizing advantages and disadvantages of each variable selection method and provide recommendations about the most appropriate choice of methods for variable selection in practice.

## Variable Selection Algorithms for Repeatedly Measured Binary Outcomes

There are several methods proposed in the literature for identifying the best subset of predictors in a GLMM or GEE setting for a repeatedly measured binary response. In this section, we describe these methods in greater detail and discuss the advantages and disadvantages of each method.

### Forward Variable Selection Algorithm

Stepwise algorithms iteratively evaluate predictors for inclusion in a regression model using forward, backward, or stepwise elimination. At each iteration, a variable is added or removed from a model based on a model performance metric such as an F-statistic, AIC, or BIC. The selection process ends when addition or removal of a variable no longer improves model fit. In traditional GLMMs, interactions are not typically included unless main effects are already in the model; thus, consideration must be given as to when interactions are considered in a model using a stepwise approach. Neerchael et al. [21] propose a stepwise selection algorithm in the GLMM setting and selects variables based on F-statistic and associated p-values. Their proposed algorithm also examines predictor interactions for inclusion or removal from the model once main effects are entered in the model. While traditional stepwise selection is commonly used in practice, there are issues with this approach [8, 22–27]. Specifically, variable subset selection is unstable in that small changes in the data can result in very different predictor subsets being selected [22, 26, 27], particularly when the number of candidate predictors is large.

### Penalized Regression for GLMM

Penalized techniques introduce a penalty term in the likelihood that penalizes regression coefficients the further they are from zero. Penalized regression methods estimate regression parameters by maximizing the penalized log-likelihood:(1)
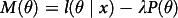
where 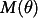 is a general objective function, 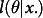 is the log-likelihood, 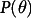 is a penalty parameter, and 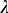 is a regularization parameter determining how much weight the penalty term carries. Common choices of 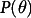 in (Eq. (1)) include the *L*_1_ penalty, 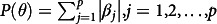, referred to as lasso regression, and the *L*_2_ penalty, 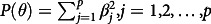, referred to as ridge regression. Unlike the *L*_2_ penalty, applying the *L*_1_ penalty yields a solution in which some of regression coefficients shrink to zero resulting in simultaneous model fitting and variable selection.

Schelldorfer et al. [12] extend lasso regression to the GLMM setting. The glmmLasso approach uses the log-likelihood function for a GLMM for 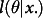 and introduces a *L*_1_ regularization parameter λ for the fix-effects vector 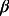 in the likelihood function. Thus, 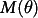 becomes *L*_1_-penalized minimizing function of the log likelihood and the glmmLasso estimator is then identified by minimizing 
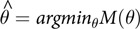
. Prior to fitting a model, the regularization parameter λ is selected by evaluating a range of possible λ’s and choosing the value that yields the minimum AIC or BIC.

### Boosting for Variable Selection in the GLMM and GEE Settings

Boosting was originally proposed to improve prediction performance of weak classifiers [13], and general boosting algorithms generate a set of models by iteratively fitting models based on a selected performance metric of the model from the previous iteration. Component-wise boosting iteratively estimates regression parameters in a linear model by maximizing the score equation based on parameter estimates from the prior iteration and then updating only the *j* ^th^ regression parameter that yields the maximum score equation [28, 29]. This approach can be used for model selection by choosing the optimal model from among models generated at each iteration based on a measure of model fit. Tutz and Groll [16] propose use of a component-wise boosting algorithm, GMMBoost, for variable selection in the GLMM setting. GMMBoost estimates the Fisher matrix and the penalized score functions for the *p* predictors at the *l* ^th^ iteration based on the intercept, the *j* ^th^ regression coefficient (*j* = 1, 2,…, *p*), and random effects from the (*l*-1)^th^ iteration. The predictor, *j*, that yields the smallest AIC or BIC is updated by adding the resulting product of the *j* ^th^ Fisher matrix and *j* ^th^ score equation to the estimates for the intercept, *j* ^th^ regression parameter and random effects from the (*l*-1)^th^ iteration. All other parameter estimates are set to the values from the (*l*-1)^th^ iteration.

The estimating equation approach has also been recently incorporated within a boosting framework for variable selection, a method referred to as EEBoost [17] and has been further extended to repeatedly measured binary outcomes [18]. Unlike the GLMM setting, there is not a closed form solution for the score equation in an estimating equation setting. Therefore, EEBoost and GEEBoost replace the score equation at each iteration with a set of estimating equations. Wolfson [17] showed that the proposed EEBoost algorithm, and by extension the GEEBoost algorithm, closely approximates the path generated from minimizing the *L*_1_-constrained projected artificial log-likelihood ratio but without necessitating a constrained optimization algorithm. The optimization for both EEBoost and GEEBoost is based on functional gradient descent optimization. GEEBoost can be used for variable selection by choosing the model from the entire path of solutions that yields the smallest QIC, although alternative criterion could also be used.

### Two-Stage Approach for Bias Correction of Parameter Estimates

Penalized and boosted regression models are known to yield biased regression parameter estimates [15, 19, 20]. We propose a two-stage approach to correct the bias in the parameter estimates from each method. Similar two-stage approaches using lasso for finding interactions have been described though the focus of is on model stability, whereas our goal is bias reduction [30]. In the first stage, a glmmLasso, GMMBoost, or GEEBoost model is fit to the data including all main effects and interactions. In the second stage, a traditional GLMM is fit using all coefficients with nonzero values in the first stage. Heredity conditions are generally used in linear models to maintain hierarchy between main effects and interactions; GlmmLasso, GMMBoost, and GEEBoost do not impose hierarchical restrictions. Under strong hierarchy, all main effects corresponding to interaction terms are included in the model [31–34]. However, there are cases where predictors are associated with an outcome through their interaction, for example, certain genetic epistasis models [35]. Thus, we considered two approaches for the two-stage GLMM: (1) impose the heredity constraint fitting a GLMM with nonzero terms from the penalized model plus all main effects that are zero in the penalized model but are in ≥1 nonzero interaction and (2) ignore the heredity constraint fitting a GLMM with only nonzero terms ignoring main effects if they are zero.

## Methods: Simulation Study

A simulation study was conducted to evaluate the ability of each method to (1) correctly recover predictors and interactions associated with a repeatedly measured binary outcome, and to evaluate (2) reduction in bias in regression parameter estimates using the two-stage approach, (3) model parsimony and (4) computational efficiency (average time to develop models). Simulation parameters including variable distributions, covariate effect size, number of observations per subject, sample size, the model used to generate the response, and the model fit using each method are defined in Table 1. Data included 14 predictors, *X*_1_-*X*_14_, yielding 91 possible two-way interactions, a random subject effect, and a repeatedly measured binary response. Correlation for time-varying covariates, Σ, was compound symmetric with *ρ* = 0.25. Potential predictors and interactions generated in the data included a mix of categorical and continuous variables as well as fixed-time and time-varying covariates to mimic what might be observed in a clinical data set. Binary response variable *Y* was generated from a Bernoulli distribution where the probability depended on variable *X*_5_, the interaction *X*_1_ *X*_2_, and the interaction *X*_2_ *X*_5_. Dependence of *Y* on the time-varying covariates *X*_1_ and *X*_2_ created a correlated response within subject. Effect sizes for nonzero parameters in the true model were selected to represent weak, moderate, and strong effects and yield odds ratios of 1.2, 2.0, and 4.5 for a one unit increase in *X*_5_, the interaction *X*_1_ *X*_2_, and the interaction *X*_2_ *X*_5_.

**Table 1.** Simulation study design

Variables	
Fixed time covariates	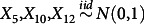
	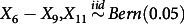
Time-varying covariates	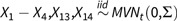 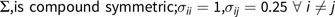
Binary response	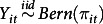
Simulation parameters	
Effect size	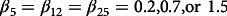 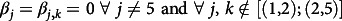
Number repeated measures	*t* = 2, 10
Sample size	*i* = 100, 200, 300, 500
Models	
Truth	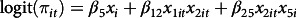
Fitted	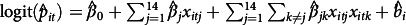

Five-hundred data sets were generated for each combination of sample and effect size for data with two observations per subject. Similarly, 500 data sets with 10 observations per subject were generated for each sample size for and effect size of 0.2. Forward stepwise GLMM, glmmLasso, GMMBoost, and GEEBoost models were fit to each simulated data set in R v 3.2.5 (Team RDC) using the *lme4*, *glmmLasso*, *GMMBoost*, and *threeboost* [36–39]. GlmmLasso, GMMBoost, and GEEboost models considered all main effects and interactions in the first stage. The forward stepwise approach [21] considered interactions for inclusion or removal from the model once main effects were entered in the model. Default settings were used to fit GMMBoost and GEEBoost models. GMMBoost and GEEBoost yield a series of models presenting the “path” by which variables enter the model, and the best model was selected based on AIC for GMMBoost and QIC for GEEBoost. GlmmLasso requires tuning of the shrinkage parameter λ and initial estimates of the fixed and random effects prior to model fitting. Shrinkage parameter and initial estimates were selected by choosing the λ that yielded the smallest AIC. Performance of each method was evaluated based on (1) the proportion of times the method correctly identified the parameters in the true underlying model and the false discovery rate (FDR) of null predictors defined as the number of null predictors selected divided by the total number of predictors selected in the model, (2) the bias = 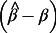 for the three predictors in the true model and the average bias for null predictors before and after using the two-stage approach, (3) model parsimony defined by the number of nonzero parameters in the final model, and (4) average computation time for model fitting. If one or more of the predictors and/or predictor interactions in the true model used to generate response *Y* where identified, the two-stage approach was applied to evaluate how well this approach reduced the bias in the parameter estimates. All simulations were conducted in R 64 bit v 3.2.5 using a Quad Opteron 2.8 GHz processor with 96 GB Ram and 3 TB of HDD storage.

## Results

### Predictor and Interaction Identification

**Fig. 1.**
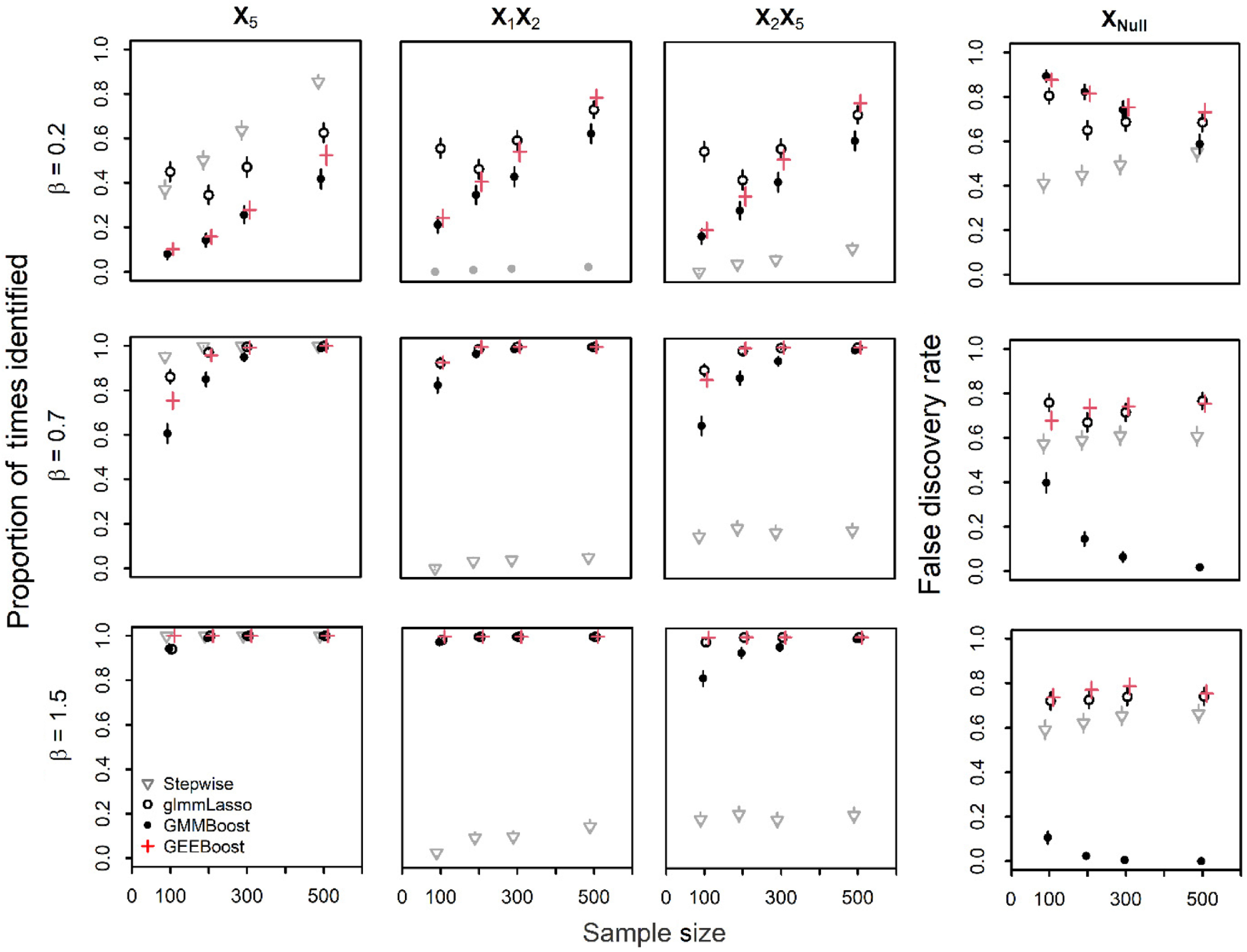
Proportion of times the true predictors, X_5_, X_1_X_2_, and X_2_X_5_ and the average false-discovery rate for null predictors (X_Null_) are selected by the four variable selection methods by effect size and sample size for data with two observations per subject.

Fig. 1 shows the proportion of times each method identifies the true predictors associated with response *Y* and the FDR for null predictors identified in data with two observations per subject for the three effect sizes and four sample sizes. Identification of the correct predictors and predictor interactions for all methods improves with increasing sample size and effect size with the exception of the stepwise selection method. The proportion of times each method correctly identifies the true predictors also increases when the number of observations per subject increases for all methods (Supplementary Fig. S1).

The stepwise algorithm correctly identifies the main effect for *X*_5_ significantly more often than the penalized regression approaches for the smallest effect size (e.g. β = 0.2) for *n* ≥ 200. However, the stepwise approach identifies the interaction terms in fewer than 20% of models across all sample sizes, effect sizes, and number of observations per subject. When sample size or effect size is small, the glmmLasso approach recovers the correct predictors and interactions more frequently than GMMBoost or GEEBoost for *n* ≤ 300. However, for large sample size or effect size (e.g. 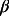 ≥ 0.7 and *n* ≥ 200), glmmLasso, GMMBoost, and GEEBoost exhibit similar ability to recover the true predictors and interactions. For *n* ≥ 200 at moderate and large effect sizes, glmmLasso, GMMBoost, and GEEBoost all recover the three predictors and predictor interactions in ≥85% of all models. The proportion of times null predictors were selected was similar within method across effect sizes. Stepwise regression and GMMBoost were less likely to select a null predictor than glmmLasso and GEEBoost. GlmmLasso was significantly more likely to select a null predictor at *n* = 100 than all other methods for 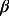 ≤ 0.7.

Among the three penalized approaches, GMMBoost tended to yield more parsimonious models relative to glmmLasso and GEEBoost for all samples and effect sizes (Table S1). GlmmLasso had the largest average number of correct predictors across simulations, though GEEBoost was similar for larger sample and effect sizes. When sample sizes and effect sizes were large, GMMBoost often exactly identified the correct model (i.e. the selected model only included *X*_5_, *X*_1_ *X*_2_, and *X*_2_ *X*_5_). In contrast, glmmLasso and GEEBoost tended to include more predictors in the model particularly for small sample sizes, which likely explains why these methods recovered the true predictors more frequently than GMMBoost. The FDR for glmmLasso and GEEBoost was between 65–81% and 68–88%, respectively (Fig. 1) and was larger for 10 observations/subject (Supplementary Fig. S1). However, FDR for GMMBoost decreased with increasing sample and effect size achieving FDR = 0 for large *n* and 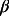. Stepwise selection had smaller FDR relative to glmmLasso and GEEBoost due to inclusion of few predictors.

### Bias and MSE of Regression Parameter Estimates

**Fig. 2.**
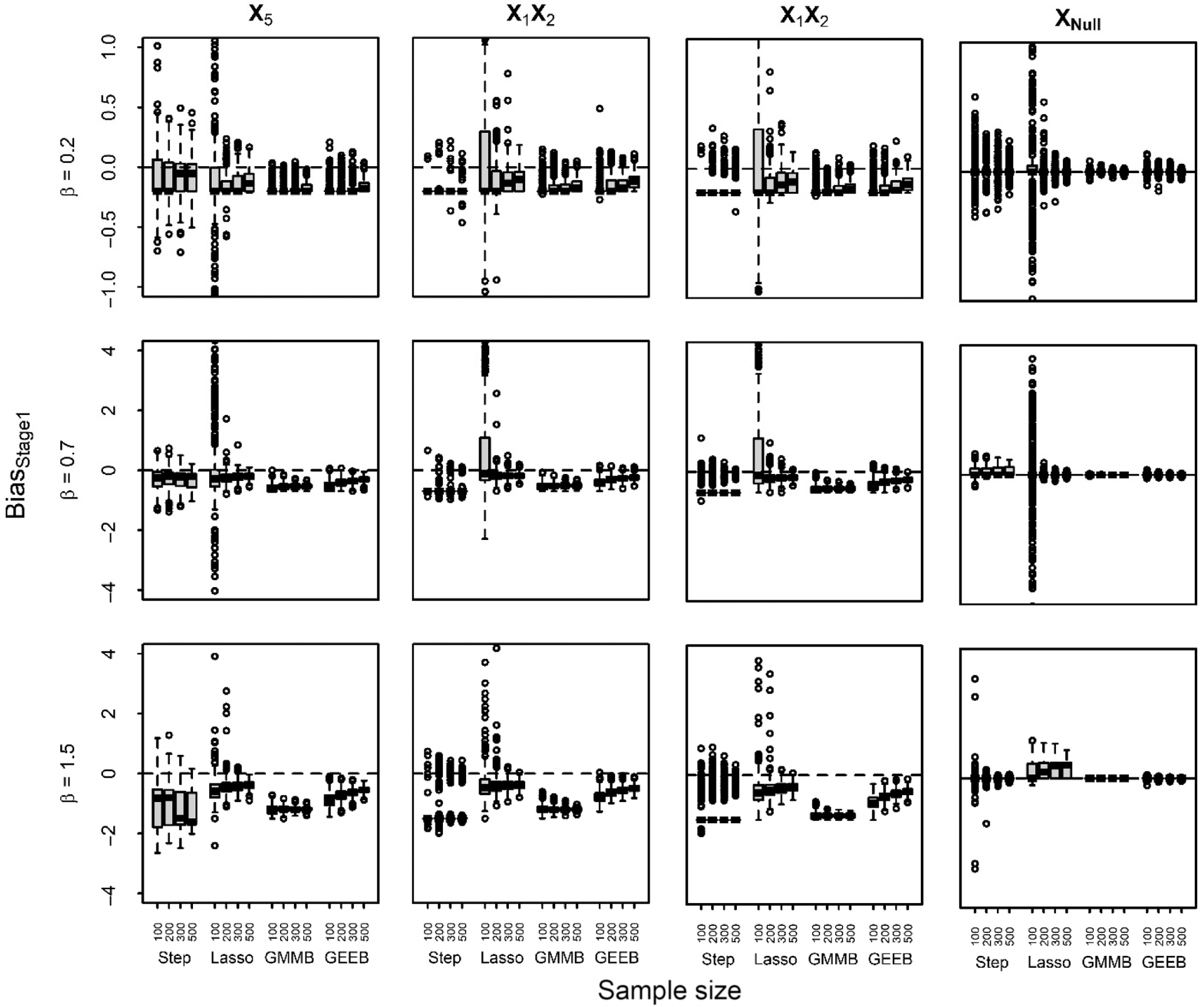
Boxplots of bias for the stage 1 models across all simulation runs for stepwise (Step), glmmLasso (Lasso), GMMBoost (GMMB), and GEEBoost (GEEB) models in data with two repeated measures per subject. Boxes represent the 25^th^, 50^th^, and 75^th^ percentiles, whiskers extend 1.5 × inner quartile range (IQR) from the 25^th^ and 75^th^ percentiles and points are values outside 1.5 × IQR. The gray dashed line indicates bias = 0.

Fig. 2 shows box plots of the bias for regression parameter estimates for *X*_5_, *X*_1_ *X*_2_, *X*_2_ *X*_5_ and the average bias across all null predictors for each approach for simulations with two observations/subject. Bias for the true predictors was generally negative indicating values smaller than the true 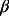. Bias decreased with increasing sample size and increased with increasing effect size across methods in data with 2 or 10 observations/subject (Figure 2; Supplementary Fig. S2 and Table S2). Stepwise selection was an exception as MSE and bias were consistent with increasing sample size (Fig. 2 and Supplementary Table S2). Among the three penalized approaches, glmmLasso yielded the smallest average bias and MSE for true predictors, followed by GEEBoost and GMMBoost (Fig. 2). The only exception for glmmLasso occurred at *n* = 100, which may result from over-fitting as average model size for glmmLasso when *n* = 100 was ~40 predictors. The stepwise approach had larger bias and MSE for X_1_X_2_ and X_2_X_5_, but similar bias to glmmLasso and GEEBoost for *X*_5_. The average bias for the 102 null predictors was small for a majority of the simulation scenarios (X_Null_, Fig. 2), though glmmLasso exhibited much larger MSE and average bias for null predictors at *n* = 100 (Supplementary Table S2). Results were similar for 10 observations/subject and effect size β = 0.2 though the observed bias was less variable (Supplementary Fig. S3, row 1).

### Two-Stage Approach for Bias Correction

**Fig. 3.**
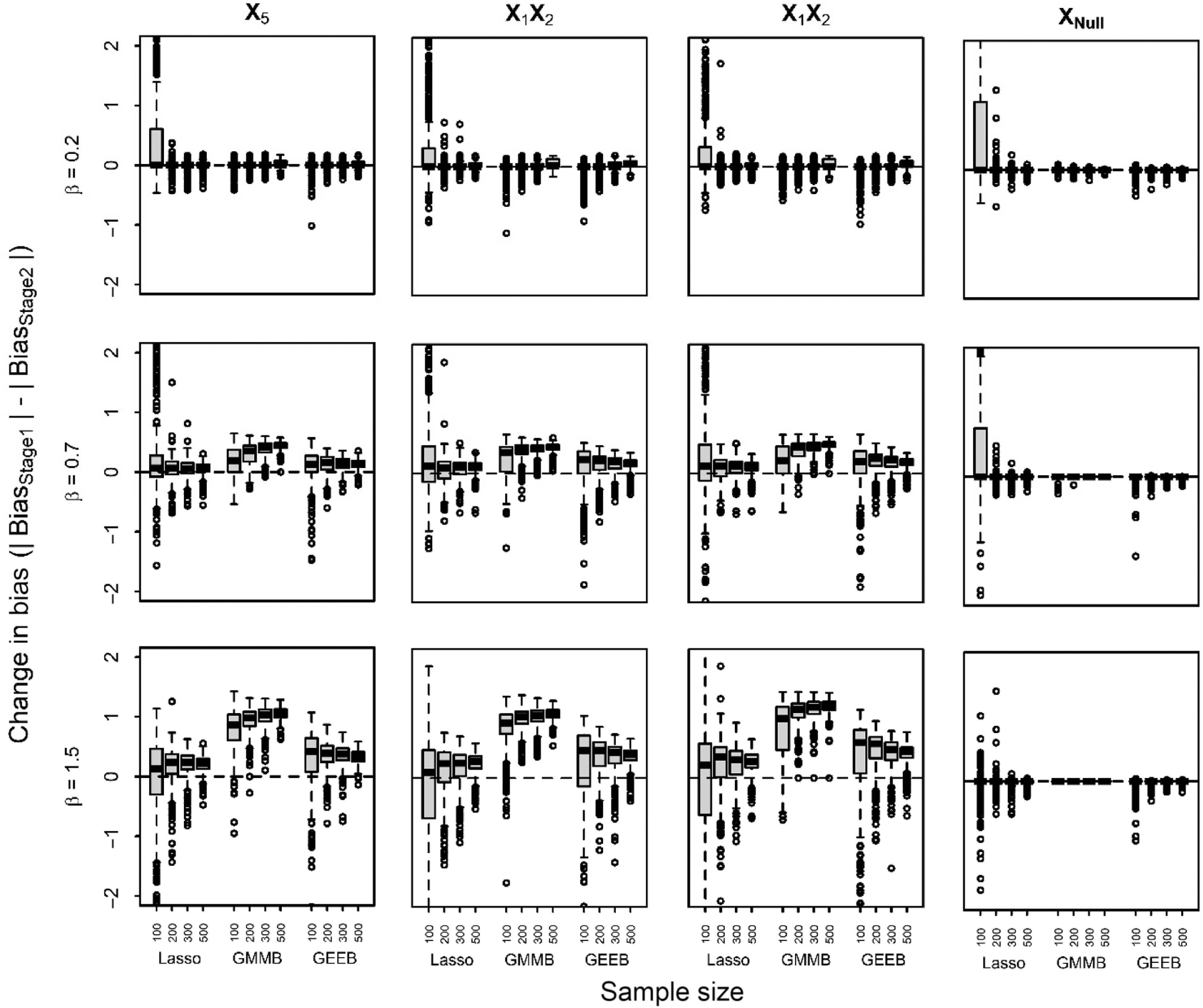
Boxplots of change in absolute bias from Stage 1 to Stage 2 bias in regression estimates for the true predictors *X*_5_, *X*_1_*X*_2_, and *X*_2_*X*_5_ and average bias for null predictors (*X*_Null_) for glmmLasso (Lasso), GMMBoost (GMMB), and GEEBoost (GEEB) for data with two measures per subject across effect and sample sizes. Boxes represent the 25^th^, 50^th^, and 75^th^ percentiles, whiskers extend 1.5 × inner quartile range (IQR) from the 25^th^ and 75^th^ percentiles, and points are values outside 1.5 × IQR. The gray dashed line indicates no difference in bias between stages.

We also evaluated the ability of a two-stage approach to reduce bias in parameters estimates using the penalized selection approaches. We considered two models: (1) the first model imposed the heredity constraint and incorporated all nonzero terms from the penalized model plus all main effects that are zero in the penalized model but are in one or more nonzero interaction term and (2) a second model in which heredity was ignored fitting a GLMM with all nonzero terms ignoring main effects if they are zero in the penalized models. Both approaches performed similarly in terms of bias reduction and thus only results for the second method are shown. The change in bias for each simulation run from stage 1 to stage 2 and the bias in stage 2 for estimates from the glmmLasso, GMMBoost, and GEEBoost in data with two observations/subject are presented in Fig. 3 and Supplementary Fig. S2, respectively.

The bias in stage 2 was generally smaller for all effect and sample sizes than in stage 1 though similar to stage 1 estimates were still predominantly downward biased (Fig. 3, an Supplementary Fig. S2). Fig. 3 demonstrates that the two-stage approach generally reduced the bias in the parameter estimates, particularly at larger effect and sample sizes. The methods had difficulty identifying the true predictors for 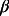 = 0.2, thus a majority of models had no change in bias in stage 2. In cases where the true predictors were selected for 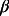 = 0.2, the bias was smaller in stage 2 relative to stage 1 for all three approaches for *n* ≥ 300. MSE was similar in the first- and second-stage models for glmmLasso and smaller for GMMBoost and GEEBoost for 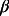 = 0.7 and 1.5 (Supplementary Table S1). GlmmLasso exhibited larger bias and MSE in the second-stage models at *n* = 100 for 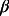 = 0.2, likely due to the large number of spurious predictors in the models. Similar trends in bias were noted for GMMBoost and GEEBoost in data with 10 observations/subject, though glmmLasso did not show decreased bias for *X*_5_ (Supplementary Fig. S3).

### Computation Time

Computation time was affected by both number of repeated measures per subject and sample size. For all methods, models fit to data with two measures/subject required less computation time compared to data with 10 measures. Of the three sparse methods, GEEBoost algorithm had the shortest computation time followed by glmmLasso and then GMMBoost. For data with two measures/subject, computation times for glmmLasso and GMMBoost were similar. However, when the number of repeated measures per subject increased to 10, GEEBoost has greatly improved computational efficiency relative to glmmLasso and GMMBoost. Average computation times for each method are presented in Supplementary Table S3.

## Results: Applications in Real Data

We applied all four variable selection approaches in data from two clinical studies, treatment response in lupus nephritis and hypothermia in total joint arthroplasty patients, to examine differences between the methods in real data applications. The two-stage approach was applied for the glmmLasso, GMMBoost, and GEEBoost models.

### Treatment Response in Lupus Nephritis

The goal of this study was to evaluate clinical and biological markers of treatment response over time in patients with lupus nephritis. Clinical and demographic information collected on 140 patients with biopsy proven lupus nephritis (LN) included age, gender, presence of anti-double-stranded DNA antibodies (dsDNA), serum C3 complement levels (C3C), serum C4 complement levels (C4C), urine protein to creatinine ratio (UrPrCr), and estimated glomerular filtration rate (EGFR). Urine samples were collected at two times during the study for all subjects and were analyzed for 15 novel urinary biomarkers. The final data include 22 demographic, clinical, or biomarker variables yielding 22 main effects and 190 two-way interactions. Patient response to treatment, determined using the criterion defined by [40] and was evaluated at 3 and 12 months after the initiation of treatment. For a full description of the study, see Ref. [41].

Predictors and interactions selected by each method for response in LN are presented in Table 2. Variables identified using stepwise algorithm are different from lasso and boosting methods. The stepwise approach selected predominantly main effects and one interaction. GlmmLasso and GMMBoost selected predominantly interactions rather than main effects. The number of predictors and interactions identified by glmmLasso and GMMBoost was similar (6 vs. 5), and there are three variables common to both methods. The regression estimates for the second-stage model are comparable between glmmLasso and GMMBoost (Table 2). GEEBoost yielded the sparsest model, including only one predictor interaction, although this interaction was also identified by glmmLasso. These results are in slight contradiction to what was observed in simulations as GMMBoost tended to give the most parsimonious model.

**Table 2.** Models of treatment response over time in patients with lupus nephritis selected by each method. Values presented for each predictor are the regression parameter estimates (standard error). Missing values indicate that the predictor was not selected in that model. Parameter estimates for the glmmLasso, GMMBoost, and GEEBoost models are from the two-stage modeling approach

	Stepwise	glmmLasso	GMMBoost	GEEBoost
AIC	269.33	270.17	267.17	279.00
No. of predictors	5	6	5	1
Intercept	−9.56 (2.01)	−2.45(0.66)	−2.12(0.40)	−5.83 (1.16)
C3C	1.72 (0.77)			
IL8	−1.74 (2.11)			
PDGFBB	4.92 (2.01)			
IL8 × PDGFBB	−7.09 (5.36)			
TWEAK	0.86 (0.44)			
IL12		0.39 (0.29)	0.50(0.22)*	
Age × NAG		−1.35 (0.38)**	−0.96(0.31)**	
C3C × TWEAK		0.31 (0.32)		
IFNα2 × IP10		0.48 (0.26)′		
IFNα2 × PDGFBB		0.28 (0.30)		1.47 (0.57)*
IL2rα × TWEAK		0.26 (0.30)	0.59(0.30)′	
Age×OPG			−0.04(0.53)	
OPG × GMCSF			−2.09(1.09)′	

***p < 0.001;** *p* < 0.01; *p < 0.05; ′*p* < 0.1.

### Hypothermia Over Time in Total Joint Arthroplasty

This was an observational study examining patient, clinical, and procedural variables associated with occurrence of hypothermia in patients undergoing total hip or total knee arthroplasty (THA and TKA). Participants’ temperatures were evaluated at eight timepoints: (1) leaving holding; (2) operating room arrival; (3) after anesthetic induction; (4) upper-body warmer initiation; (5) incision; (6) every 30 min after incision; (7) leaving the operating room; and (8) arrival to PACU and hypothermia was defined as temperature < 36.0°C. The data included 102 participants with 13 demographic, clinical, or procedural variables and 66 possible two-way interactions. Full study details are provided in Ref. [42].

Similar to the lupus nephritis study, the model selected using forward selection was quite different from the models selected using the penalized regression and boosting approaches. The forward selection model only included main effects, while the other approaches included a mix of main effects and interactions. The glmmLasso model included more predictors than GMMBoost and GEEBoost (18 vs. 4 or 6, respectively; Table 3). Although glmmLasso included the largest number of predictors, three of four predictors identified by GMMBoost and four of six predictors selected by GEEBoost were also selected by glmmLasso. The glmmLasso, GMMBoost, and GEEBoost models had three terms in common: the interaction between BMI and gender, between procedure type and anesthesia type, and between procedure type and phenylephrine dose. Regression estimates for these terms were of similar magnitude between the methods although only the interaction between procedure type and phenylephrine dose was statistically significant for three methods, while gender by BMI was only significant in the GMMBoost and GEEBoost models, and procedure type by anesthesia type was not significant in any model.

**Table 3.** Models of incidence of hypothermia over time in patients undergoing total joint arthroplasty selected by each method. Values presented for each predictor are the regression parameter estimates (standard error). Missing values indicate that the predictor was not selected in that model. Parameter estimates for the glmmLasso, GMMBoost, and GEEBoost models are from the two-stage modeling approach

	Stepwise	glmmLasso	GMMBoost	GEEBoost
AIC	777.47	783.44	774.70	774.30
# predictors	4	18	4	6
Intercept	−2.86 (0.54)	−5.57 (3.10)	−2.01 (0.26)	−1.97 (0.27)
Cumulative time		−0.14 (0.10)		−0.11 (0.10)
Age		0.74 (0.61)		
Sex (Males vs. Female)	−1.20 (0.38)			
Anesthesia type (Spinal vs. General)	1.16 (0.51)	4.65 (3.61)		
Procedure (TKA vs. THA)	1.10 (0.36)			
Body Mass Index (BMI)	−0.45 (0.18)			
Operating Room (OR) Temperature		−0.20 (0.25)		
Age × Sex		−0.49 (0.69)		
Age × Procedure		0.15 (0.47)		
Age × Anesthesia Type		−1.32 (1.41)		0.32 (0.20)
Sex × BMI		−0.15 (0.67)	−0.50 (0.19)**	−0.58 (0.19)**
Sex × Revision (Yes/No)		−0.64 (0.97)		
Sex × Procedure duration		0.22 (0.67)		
BMI × Revision		−0.17 (0.32)		
BMI × OR temperature		−0.28 (0.19)		
BMI × Total IV fluid			−0.33 (0.22)	
BMI × OR humidity				−0.22 (0.17)
Procedure × Phenylephrine Dose		0.62 (0.20)**	0.39 (0.17)*	0.46 (0.17)**
Procedure × Anesthesia Type		−0.06 (0.98)	0.60 (0.11)	0.49 (0.41)
Revision × Total IV Fluid		0.01 (0.40)		
Phenylephrine Dose × Anesthesia Type		−0.46 (0.28)		
Mattress Temperature × OR Temperature		−0.05 (0.28)		
OR Temperature × OR Humidity		−0.14 (0.16)		

***p < 0.001; **p < 0.01; *p < 0.05; ′*p* < 0.1.

## Discussion

Identifying predictors and predictor interactions associated with repeatedly measured binary disease outcomes is important as many common diseases are hypothesized to result from interactions between patient, clinical, and environmental factors. Statistical methods for variable selection, including penalized and boosted regression approaches, have recently been developed for repeatedly measured binary outcomes. This study compared the ability of four variable selection techniques, forward stepwise selection, glmmLasso, GMMBoost, and GEEBoost, to correctly recover predictors and interactions associated with a repeatedly measured binary outcome to provide guidance for choosing an appropriate method in application. Additionally, we evaluated a two-stage approach to reduce bias for regression parameter estimates selected by glmmLasso, GMMBoost, or GEEBoost. Our simulations corroborate previous findings regarding the poor performance of the forward stepwise approach [9, 10] and showed the effectiveness of boosting and penalized regression techniques while demonstrating differences between the approaches.

The boosting and penalized regression approaches demonstrated superior recovery of the correct predictors relative to the forward stepwise selection approach. Among the penalized and boosting regression approaches, glmmLasso recovered the correct model terms more frequently than the other approaches, particularly at smaller effect and sample sizes. However, glmmLasso models often included many spurious terms yielding a high FDR. GEEBoost performed similarly to glmmLasso for predictor recovery and generated slightly more parsimonious models though FDR was also large. GMMBoost had greater difficulty recovering the correct terms; however, it returned the most parsimonious model, often selecting the exact model used to generate the underlying response. The models selected in the two applications were consistent with the simulations in that glmmLasso selected the largest number of predictors followed by GEEBoost and then GMMBoost. In terms of computation, GEEBoost had the fastest computation time followed by glmmLasso and GMMBoost. Of note, GMMBoost had much greater computation time relative to the other two methods at larger sample sizes, so much so that we were unable to conduct simulations for GMMBoost with sample size *n* = 500 and 10 observations per subject.

The penalized and boosting regression approaches almost always yielded regression parameter estimates biased toward zero. The only exceptions occurred for glmmLasso at *n* = 100, where this approach had difficulty selecting a reduced subset of predictors. In all simulations, using a two-stage approach effectively reduced the bias for all three methods regardless of sample sizes, effect sizes, or observations/subject.

The simulations provide guidance for choosing an appropriate method to identify predictor interactions without *a priori* knowledge in data with a repeatedly measured binary outcome. Table 4 provides suggestions for choosing among glmmLasso, GMMBoost, and GEEBoost based on study goals and computing efficiency. The glmmLasso approach recovered the true regression parameters most often but produced the least parsimonious models; thus, if the goal is to select candidate predictors and interactions with assurance that relevant parameters will be selected, then glmmLasso is a most appropriate given sufficiently large sample size (*n* ≥ 200). GMMBoost recovered the correct predictors and interactions with similar frequency to glmmLasso and GEEBoost at large effect and sample sizes and identified few spurious predictors; thus, GMMBoost is a most appropriate if parsimony is of importance. GMMBoost is the least computationally efficient and may prove difficult in real applications. At smaller sample sizes, GEEBoost had similar performance to glmmLasso and tended to select a more parsimonious model thus offering a good compromise between the computation time and model parsimony. Finally, the two-stage approach effectively reduced the bias for all three methods and thus should be applied regardless of selected method.

**Table 4.** Guidance for selecting the optimal variable selection method in data with a repeated binary outcome

Model parsimony	Predictor/ interaction identification	Computational efficiency	Method
Moderate importance	Highly important	Moderate	glmmLasso
Highly important	Moderate to high importance	Limited	GMMBoost
Moderate to high importance	Moderate to high importance	Excellent	GEEBoost

There are extensions that could be considered to enhance the applicability of each method. Elastic net uses both *L*_*1*_ and *L*_*2*_ constraints to yield a sparse solution while facilitating grouping of collinear variables [43]. Additionally, several penalized regression approaches for data with independent observations exist that impose heredity constraints to maintain hierarchy between main effects and interactions [33, 44, 45]. These approaches improve model error rate and to yield more parsimonious models relative to traditional lasso. Given the large FDR observed for glmmLasso and GEEBoost, extending these approaches to allow for grouping or hierarchy would likely yield more parsimonious models and reduce FDR.

## Conclusion

In this paper, we describe four variable selection approaches for data with repeatedly measured binary outcomes, with focus on identifying predictor interactions without an *a priori* hypothesis. Our results demonstrate a strong advantage of penalized and boosted approaches over the traditional stepwise approach in these types of data and provide guidance for choosing the most appropriate method in real applications. Furthermore, the two-stage approach effectively reduces the bias and MSE for all methods and should be applied regardless of choice of method.

## Supplementary material

For supplementary material accompanying this paper visit https://doi.org/10.1017/cts.2020.556. click here to view supplementary material
