# Supplementary material for: Variable selection methods for identifying predictor interactions in data with repeatedly measured binary outcomes
Source: J Clin Transl Sci. 2020 Nov 16;5(1):e59. doi: 10.1017/cts.2020.556 (PMC8057419; doi:10.1017/cts.2020.556)
Supplement: Supplementary file 1 [file S2059866120005567sup001.pdf]

Supplementary Materials: Variable selection methods for identifying predictor interactions in data with repeatedly measured binary outcomes

Table S1: Mean (SD) for the number of non-zero predictors selected by each approach in Stage 1. All = total number of non-zero predictors selected; Correct = number of true predictors (X_5, X_1X_2, and X_2X_5) selected; Null = number of null parameters selected. The effect size is the expected β for the true predictors.

Effect Size	Obs/Subject	N	Predictors	Stepwise	glmmLasso	GMMBoost	GEEBoost
0.2	2	100	All	1.84 (1.94)	41.9 (45.8)	4.54 (1.25)	5.96 (6.95)
			Correct	0.38 (0.5)	1.56 (1.26)	0.46 (0.61)	0.54 (0.75)
			Null	1.46 (1.71)	40.35 (44.7)	4.08 (1.33)	5.42 (6.54)
		200	All	2.51 (2.65)	10.17 (14.14)	4.65 (1.18)	7.32 (7.74)
			Correct	0.56 (0.59)	1.23 (1.06)	0.77 (0.68)	0.91 (0.84)
			Null	1.95 (2.34)	8.93 (13.57)	3.88 (1.32)	6.4 (7.3)
		300	All	2.92 (2.63)	10.57 (10.14)	4.62 (1.18)	8.51 (8.11)
			Correct	0.71 (0.61)	1.63 (0.99)	1.1 (0.77)	1.33 (0.94)
			Null	2.21 (2.32)	8.94 (9.63)	3.52 (1.44)	7.18 (7.61)
		500	All	3.6 (2.67)	10.64 (8.95)	4.34 (1.2)	11.56 (8.33)
			Correct	0.99 (0.56)	2.07 (0.92)	1.64 (0.75)	2.08 (0.86)
			Null	2.61 (2.4)	8.57 (8.56)	2.7 (1.44)	9.48 (7.93)
	10	100	All	2.35 (1.88)	11.97 (10.5)	5.31 (2.12)	33.92 (17.55)
			Correct	0.66 (0.58)	1.94 (0.82)	1.49 (0.72)	2.2 (0.67)
			Null	1.69 (1.58)	10.03 (10.07)	3.81 (2.04)	31.72 (17.26)
		200	All	3.22 (2.42)	30.13 (18.09)	0.03 (0.36)	38.71 (18.19)
			Correct	0.95 (0.62)	2.69 (0.47)	0.01 (0.15)	2.58 (0.51)
			Null	2.26 (2.08)	27.43 (17.99)	0.02 (0.21)	36.13 (18.08)
		300	All	3.68 (2.42)	49.77 (26.82)	*	40.41 (19.05)
			Correct	1.1 (0.55)	2.89 (0.32)	*	2.72 (0.45)
			Null	2.58 (2.2)	46.89 (26.78)	*	37.68 (18.99)
		500	All	3.7 (2.31)	88.5 (9.57)	*	41.63 (16.99)
			Correct	1.16 (0.45)	2.95 (0.21)	*	2.89 (0.32)
			Null	2.54 (2.14)	85.55 (9.55)	*	38.74 (16.99)
0.7	2	100	All	3.36 (1.54)	40.17 (41.02)	3.7 (1.05)	11.83 (7.53)
			Correct	1.1 (0.44)	2.69 (0.73)	2.08 (0.67)	2.54 (0.64)
			Null	2.26 (1.41)	37.48 (40.78)	1.62 (1.16)	9.3 (7.27)
		200	All	4.01 (2.38)	16.07 (13.04)	3.24 (0.73)	15.26 (7.98)
			Correct	1.22 (0.5)	2.95 (0.28)	2.68 (0.49)	2.95 (0.21)
			Null	2.79 (2.18)	13.13 (13)	0.56 (0.73)	12.3 (7.97)
		300	All	4 (2.37)	16.74 (10.87)	3.13 (0.52)	16.01 (8.84)
			Correct	1.21 (0.5)	2.99 (0.08)	2.88 (0.33)	2.99 (0.09)
			Null	2.79 (2.18)	13.75 (10.87)	0.25 (0.5)	13.02 (8.85)
		500	All	4.1 (2.49)	18.3 (10.59)	3.05 (0.27)	16.65 (8.78)
			Correct	1.23 (0.53)	3 (0)	2.98 (0.15)	3 (0)
			Null	2.87 (2.3)	15.3 (10.59)	0.07 (0.26)	13.65 (8.78)
1.5	2	100	All	3.59 (1.41)	18.89 (17.33)	3.14 (0.72)	14.08 (5.68)
			Correct	1.21 (0.46)	2.9 (0.42)	2.73 (0.47)	3 (0.04)
			Null	2.38 (1.32)	15.98 (17.27)	0.41 (0.61)	11.08 (5.68)
		200	All	4.37 (2.4)	18.93 (14.17)	3.01 (0.38)	15.87 (6.32)
			Correct	1.3 (0.63)	3 (0)	2.92 (0.27)	3 (0)
			Null	3.07 (2.13)	15.93 (14.17)	0.09 (0.29)	12.87 (6.32)
		300	All	4.65 (2.6)	18.63 (11.63)	2.98 (0.26)	17.17 (6.9)
			Correct	1.28 (0.63)	3 (0)	2.96 (0.21)	3 (0)
			Null	3.38 (2.31)	15.63 (11.63)	0.02 (0.15)	14.17 (6.9)
		500	All	4.81 (2.6)	18.46 (10.74)	2.99 (0.09)	16.65 (8.78)
			Correct	1.35 (0.72)	3 (0)	2.99 (0.09)	3 (0)
			Null	3.47 (2.29)	15.46 (10.74)	0 (0)	13.65 (8.78)

***** GMMBoost was not run for data with 10 observations per subject at n = 300 or n = 500 due to excessive computing time.

Table S2: Mean squared error (MSE) and average bias for $\hat{\beta}$ estimates for variables X_5, X_1X_2, and X_2X_5 and the range of MSE and bias for null predictors (X_{Null}) from stage 1 and stage 2 models in data with 2 observations per subject. Second stage models are only fit for penalized regression approaches.

β	Model	N	X_5				X_1X_2				X_2X_5				X_{Null}			
			**Stage 1**		**Stage 2**		**Stage 1**		**Stage 2**		**Stage 1**		**Stage 2**		**Stage 1**		**Stage 2**	
			**MSE**	**Bias**	**MSE**	**Bias**	**MSE**	**Bias**	**MSE**	**Bias**	**MSE**	**Bias**	**MSE**	**Bias**	**MSE**	**Bias**	**MSE**	**Bias**
		100	0.058	-0.102	-	-	0.040	-0.199	-	-	0.040	-0.198	-	-	0, 0.019	0, 0.006	-	-
	Stepwise	200	0.035	-0.090	-	-	0.040	-0.196	-	-	0.039	-0.188	-	-	0, 0.012	0, 0.024	-	-
		300	0.030	-0.079	-	-	0.040	-0.196	-	-	0.038	-0.185	-	-	0, 0.008	0, 0.011	-	-
		500	0.024	-0.066	-	-	0.040	-0.197	-	-	0.036	-0.177	-	-	0, 0.007	0, 0.024	-	-
		100	2.309	0.021	0.043	-0.162	0.918	0.214	0.060	-0.107	1.301	0.275	0.056	-0.119	6.602, 261.4	-2.68, 1.55	182, 7195	-7.81, 5.58
	glmmLasso	200	0.033	-0.153	0.036	-0.121	0.043	-0.107	0.034	-0.063	0.038	-0.127	0.033	-0.080	0.002, 0.229	-0.160, 0.344	0.003, 0.389	-0.028, 0.059
		300	0.026	-0.132	0.029	-0.092	0.024	-0.109	0.021	-0.053	0.023	-0.111	0.024	-0.055	0.001, 0.051	-0.013, 0.084	0.001, 0.092	-0.007, 0.052
0.2		500	0.022	-0.118	0.022	-0.068	0.017	-0.100	0.014	-0.038	0.018	-0.102	0.016	-0.040	0.000, 0.020	-0.005, 0.030	0.000, 0.052	-0.006, 0.037
		100	0.038	-0.192	0.041	-0.169	0.034	-0.176	0.051	-0.103	0.036	-0.183	0.048	-0.125	0.000, 0.060	-0.002, 0.015	0.000, 0.661	-0.010, 0.091
	GMMBoost	200	0.036	-0.186	0.037	-0.158	0.032	-0.168	0.035	-0.083	0.034	-0.178	0.035	-0.109	0.000, 0.012	-0.002, 0.011	0.000, 0.090	-0.006, 0.076
		300	0.034	-0.181	0.032	-0.141	0.030	-0.167	0.027	-0.084	0.031	-0.167	0.028	-0.085	0.000, 0.008	-0.000, 0.001	0.000, 0.110	-0.006, 0.095
		500	0.031	-0.171	0.026	-0.114	0.027	-0.155	0.017	-0.055	0.027	-0.158	0.019	-0.058	0.000, 0.001	-0.000, 0.006	0.000, 0.127	-0.005, 0.103
		100	0.037	-0.188	0.046	-0.165	0.034	-0.167	0.089	-0.077	0.034	-0.172	0.058	-0.102	0.013, 2.063	-0.842, 6.374	0.001, 0.179	-0.012, 0.097
	GEEBoost	200	0.036	-0.185	0.038	-0.159	0.028	-0.152	0.035	-0.064	0.031	-0.164	0.035	-0.089	0.001, 0.156	-0.044, 0.298	0.001, 0.125	-0.013, 0.087
		300	0.033	-0.176	0.032	-0.142	0.025	-0.146	0.023	-0.058	0.026	-0.146	0.026	-0.059	0.000, 0.076	-0.006, 0.087	0.001, 0.164	-0.007, 0.121
		500	0.028	-0.159	0.024	-0.111	0.018	-0.118	0.012	-0.027	0.019	-0.122	0.013	-0.029	0.000, 0.041	-0.003, 0.037	0.000, 0.152	-0.009, 0.128
		100	0.278	-0.338	-	-	0.490	-0.698	-	-	0.428	-0.608	-	-	0, 0.082	0, 0.097	-	-
	Stepwise	200	0.200	-0.296	-	-	0.485	-0.687	-	-	0.405	-0.586	-	-	0, 0.070	0, 0.094	-	-
		300	0.220	-0.334	-	-	0.480	-0.682	-	-	0.412	-0.597	-	-	0, 0.121	0, 0.075	-	-
		500	0.206	-0.342	-	-	0.479	-0.682	-	-	0.407	-0.595	-	-	0, 0.126	0, 0.075	-	-
		100	3.083	0.138	0.534	-0.276	3.799	0.683	1.015	-0.031	4.419	0.722	0.757	-0.074	6.306, 249.1	-6.621, 8.793	145, 5735	-4.41, 5.86
	glmmLasso	200	0.114	-0.260	0.097	-0.116	0.082	-0.176	0.056	0.040	0.086	-0.223	0.055	0.014	0.004, 0.031	-0.061, 0.185	0.006, 0.051	-0.012, 0.038
		300	0.072	-0.217	0.061	-0.089	0.050	-0.178	0.023	0.021	0.057	-0.199	0.025	0.010	0.001, 0.014	-0.038, 0.130	0.002, 0.026	-0.010, 0.034
0.7		500	0.053	-0.200	0.038	-0.093	0.042	-0.181	0.013	-0.013	0.046	-0.191	0.014	-0.013	0.001, 0.007	-0.051, 0.143	0.002, 0.012	-0.008, 0.021
		100	0.362	-0.589	0.231	-0.332	0.299	-0.530	0.124	-0.089	0.354	-0.580	0.211	-0.190	0, 0.014	-0.001, 0.012	0, 0.234	-0.006, 0.122
	GMMBoost	200	0.307	-0.543	0.111	-0.174	0.274	-0.515	0.039	-0.034	0.332	-0.568	0.087	-0.093	0, 0.001	-0.000, 0.001	0, 0.082	-0.004, 0.063
		300	0.295	-0.536	0.053	-0.094	0.266	-0.510	0.020	-0.023	0.324	-0.564	0.045	-0.054	0, 0.001	0, 0.001	0, 0.032	-0.001, 0.025
		500	0.284	-0.529	0.019	-0.053	0.271	-0.518	0.011	-0.039	0.320	-0.562	0.016	-0.041	0, 0	0, 0.001	0, 0.008	0, 0.006
		100	0.299	-0.524	1.348	-0.240	0.194	-0.409	1.312	0.193	0.240	-0.454	2.665	0.163	0.025, 3.050	-0.575, 2.153	0.004, 0.532	-0.045, 0.168

	GEEBoost	200	0.188	-0.407	0.121	-0.212	0.110	-0.305	0.043	0.047	0.137	-0.343	0.042	0.030	0.002, 0.056	-0.042, 0.195	0.003, 0.084	-0.018, 0.083
		300	0.146	-0.361	0.084	-0.189	0.082	-0.267	0.021	0.023	0.101	-0.298	0.023	0.014	0.001, 0.019	-0.039, 0.174	0.002, 0.036	-0.014, 0.062
		500	0.105	-0.307	0.050	-0.152	0.066	-0.242	0.012	-0.013	0.076	-0.262	0.012	-0.011	0.000, 0.011	-0.029, 0.147	0.001, 0.018	-0.008, 0.040
		100	1.528	-1.038	-	-	2.225	-1.476	-	-	1.920	-1.310	-	-	0, 0.318	0, 0.282	-	-
	Stepwise	200	1.486	-1.037	-	-	2.156	-1.424	-	-	1.827	-1.241	-	-	0, 0.509	0, 0.171	-	-
		300	1.785	-1.197	-	-	2.086	-1.387	-	-	1.876	-1.275	-	-	0, 0.631	0, 0.118	-	-
		500	1.887	-1.256	-	-	2.032	-1.354	-	-	1.818	-1.238	-	-	0, 0.751	0, 0.079	-	-
		100	1.234	-0.477	4.116	0.176	2.154	-0.229	16.051	0.788	1.339	-0.423	9.643	0.630	0.513, 34.00	-1.623, 1.631	7.69, 509.1	-1.20, 1.20
	glmmLasso	200	0.288	-0.433	0.169	-0.054	0.271	-0.394	0.169	0.134	0.350	-0.461	0.179	0.105	0.008, 0.093	-0.336, 0.908	0.016, 0.178	-0.033, 0.090
		300	0.210	-0.419	0.113	-0.057	0.184	-0.381	0.142	0.099	0.228	-0.431	0.129	0.085	0.004, 0.023	-0.169, 0.478	0.009, 0.047	-0.021, 0.060
1.5		500	0.159	-0.376	0.048	-0.084	0.161	-0.376	0.029	0.004	0.183	-0.398	0.032	0.002	0.002, 0.010	-0.210, 0.454	0.004, 0.019	-0.017, 0.037
		100	1.481	-1.206	0.369	-0.195	1.426	-1.185	0.297	-0.002	1.846	-1.353	0.579	-0.197	0, 0	0, 0	0.000, 0.107	-0.004, 0.085
	GMMBoost	200	1.396	-1.176	0.107	-0.076	1.434	-1.194	0.060	-0.034	1.841	-1.354	0.226	-0.105	0, 0	0, 0	0, 0.026	-0.002, 0.025
		300	1.412	-1.185	0.051	-0.044	1.435	-1.195	0.050	-0.027	1.823	-1.348	0.144	-0.075	0, 0	0, 0	0, 0.005	0, 0.006
		500	1.410	-1.186	0.025	-0.055	1.441	-1.199	0.025	-0.056	1.819	-1.348	0.046	-0.058	0, 0	0, 0	0, 0	0, 0
		100	0.839	-0.887	4.278	0.155	0.640	-0.773	7.739	0.885	0.855	-0.900	6.184	0.756	0.010, 2,471	-0.520, 2.353	0.008, 2.007	-0.073, 0.329
	GEEBoost	200	0.545	-0.714	0.196	-0.202	0.426	-0.632	0.116	0.139	0.552	-0.724	0.119	0.096	0.002, 0.071	-0.128, 0.556	0.004, 0.098	-0.032, 0.140
		300	0.418	-0.629	0.116	-0.168	0.324	-0.549	0.081	0.082	0.406	-0.620	0.073	0.062	0.001, 0.029	-0.082, 0.437	0.002, 0.058	-0.019, 0.102
		500	0.313	-0.546	0.073	-0.165	0.254	-0.490	0.027	0.002	0.312	-0.544	0.033	-0.001	0.001, 0.013	-0.091, 0.468	0.001, 0.018	-0.008, 0.040

Table S3: Average computation time in minutes for each methods by number of observations per subject and sample size.

Method	Observations/Subject	N	Computation Time (min)
glmmLasso	2	100	1.37
		200	4.39
		300	6.99
		500	35.29
	10	100	3.11
		200	15.41
		300	43.65
		500	246.905
GMMBoost	2	100	0.69
		200	4.42
		300	14.04
		500	62.42
	10	100	37.95
		200	406.1
		300	1394.13
		500	
GEEBoost	2	100	1.44
		200	5.54
		300	12.57
		500	36.43
	10	100	2.26
		200	7.25
		300	17.84
		500	45.22

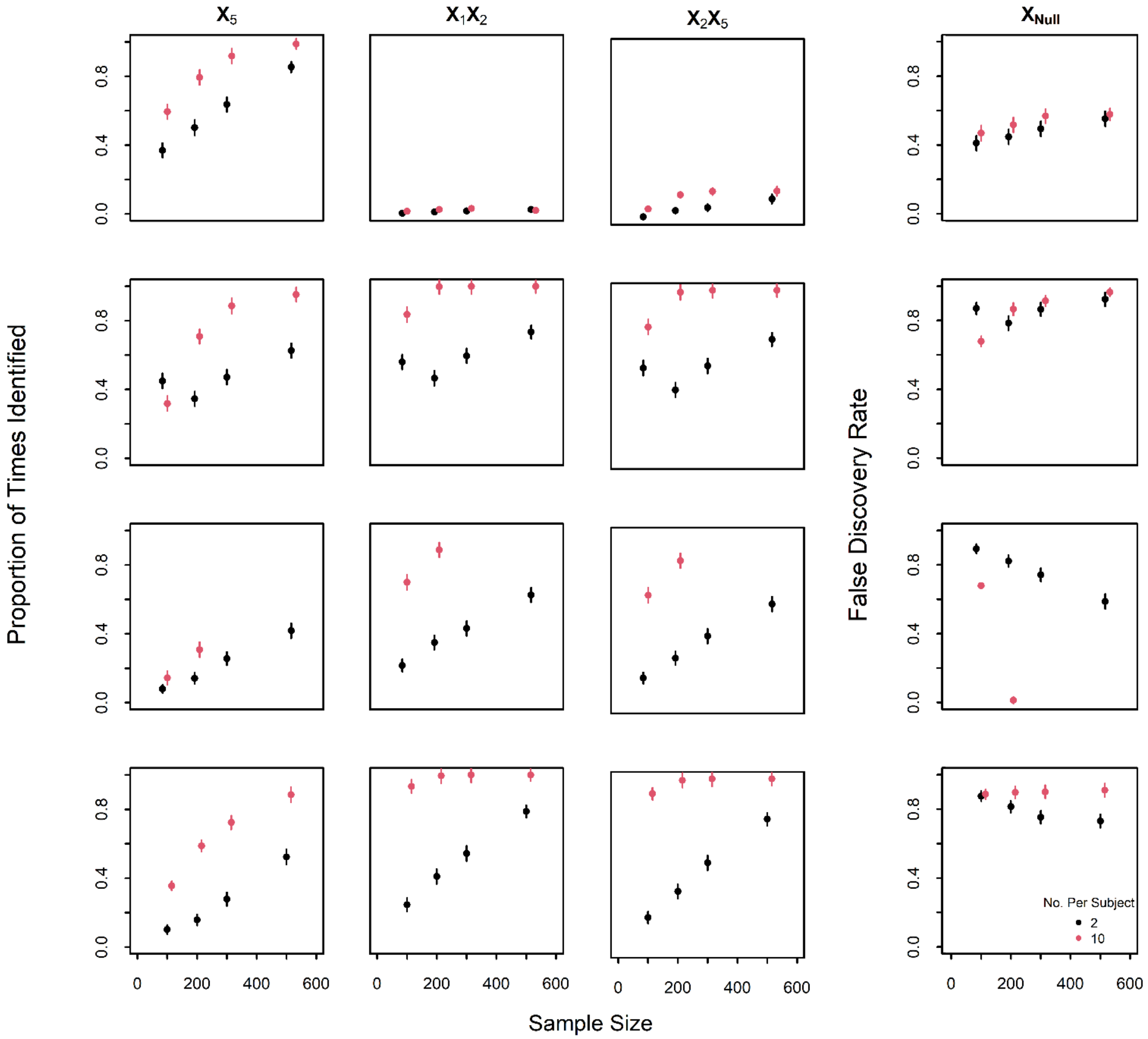

Figure S1: Proportion of times the true predictors, X_5, X_1X_2, and X_2X_5 and the average FDR for null predictors (X_{Null}) selected by each methods for simulated data with 2 or 10 repeated measures per subject in data with effect size = 0.2 as sample size increases. Black circles represent the proportion for data with 2 measures/subject and red circles represent the proportion for 10 measures/subject.

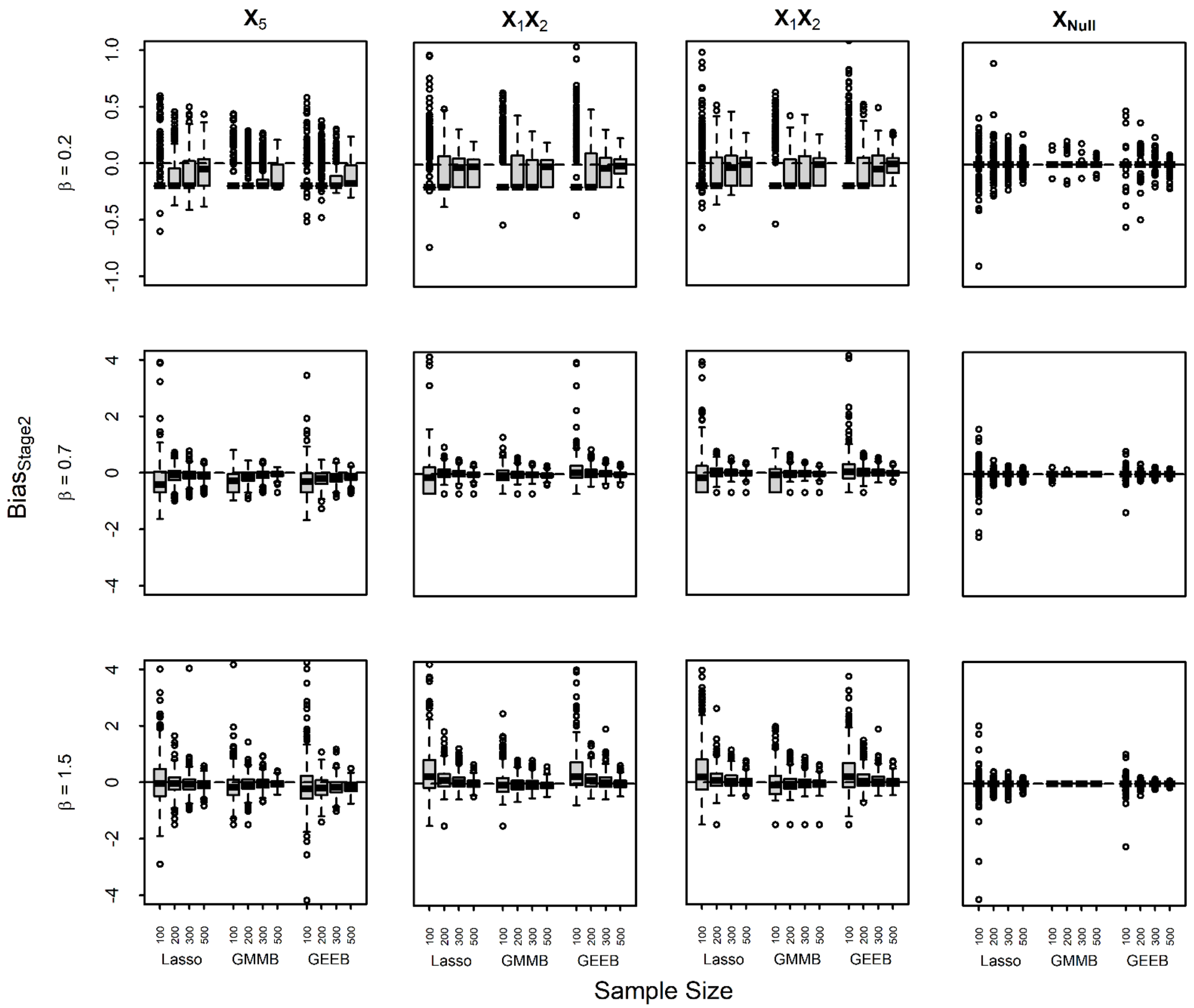

Figure S2: Boxplots of bias for the stage 2 models across all simulation runs for glmmLasso (Lasso), GMMBoost (GMMB), and GEEBoost (GEEB) models in data with 2 repeated measures per subject. Boxes represent the 25th, 50th, and 75th percentiles, whiskers extend 1.5*inner quartile range (IQR) from the 25th and 75th percentiles and points are values outside 1.5*IQR. The grey dashed line indicates bias = 0.

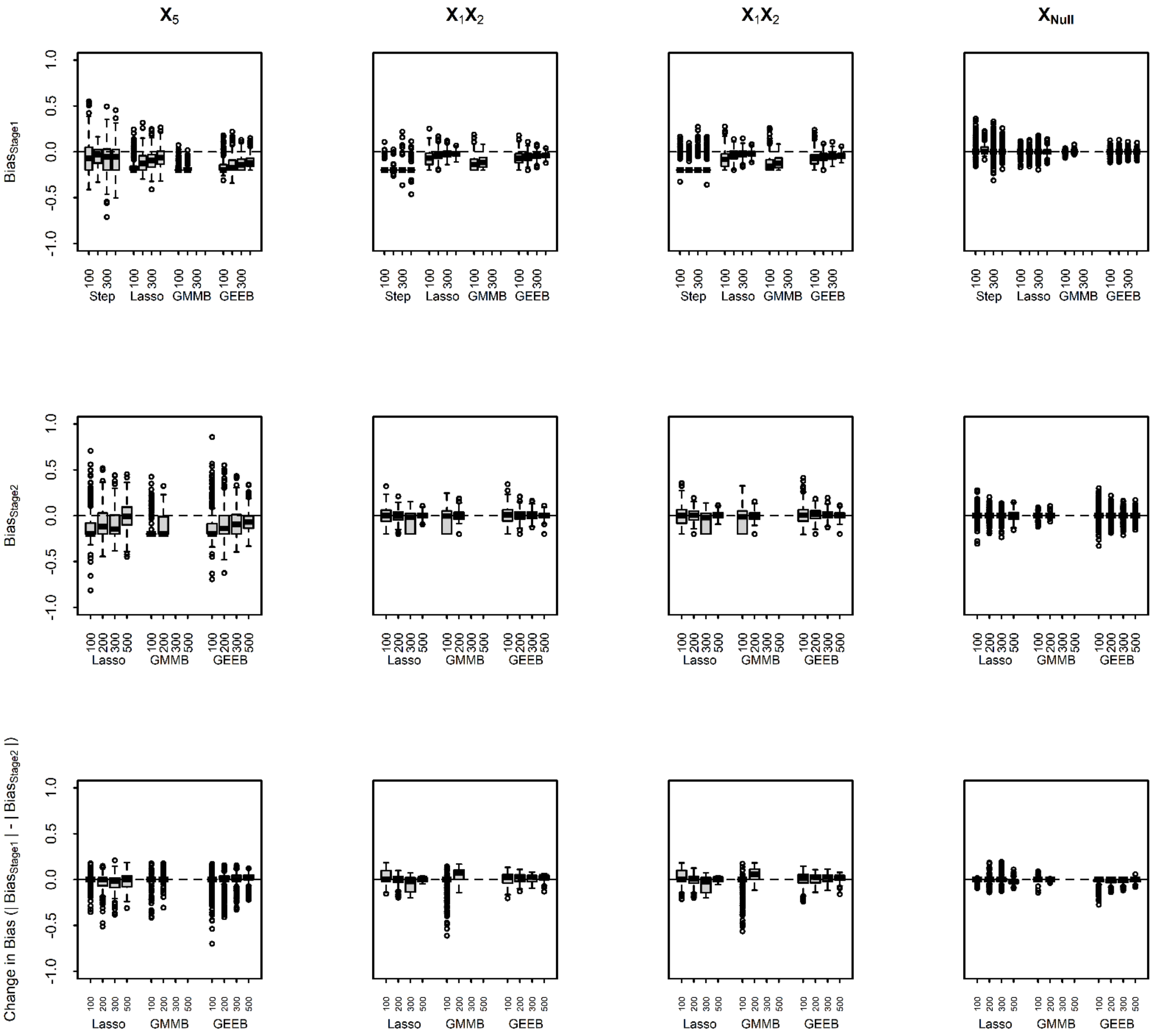

Figure S3: Boxplots of bias for the stage 1, stage 2, and the change in bias from stage 1 to stage 2 across all simulation runs forstepwise (Step), glmmLasso (Lasso), GMMBoost (GMMB), and GEEBoost (GEEB) models in data with 10 repeated measures per subject. Note, stepwise models are fitted in a single stage and therefore are only represented for stage 1 bias. Also GMMBoost models with 300 and 500 observations were not run due to computational constraints. Boxes represent the 25th, 50th, and 75th percentiles, whiskers extend 1.5*inner quartile range (IQR) from the 25th and 75th percentiles and points are values outside 1.5*IQR. The grey dashed line indicates bias = 0.
